# Influence of Plasma Treatment on the Polyphenols of Food Products—A Review

**DOI:** 10.3390/foods9070929

**Published:** 2020-07-14

**Authors:** Paulo E. S. Munekata, Rubén Domínguez, Mirian Pateiro, José M. Lorenzo

**Affiliations:** 1 Centro Tecnolóxico da Carne de Galicia, rúa Galicia No 4, Parque Tecnolóxico de Galicia, San Cibrao das Viñas, 32900 Ourense, Spain; paulosichetti@ceteca.net (P.E.S.M.); rubendominguez@ceteca.net (R.D.); mirianpateiro@ceteca.net (M.P.); 2 Área de Tecnología de los Alimentos, Facultad de Ciencias de Ourense, Universidad de Vigo, 32004 Ourense, Spain

**Keywords:** phenolic compounds, nonthermal processing, radicals, fruits, juice, seeds

## Abstract

The consumption of bioactive compounds, especially phenolic compounds, has been associated with health benefits such as improving the health status and reducing the risk of developing certain diseases such as cancer, cardiovascular diseases, and neurodegenerative disorders. However, the preservation of natural bioactive compounds in food products is a major challenge for the food industry. Due to the major impact of conventional thermal processing, nonthermal technologies such as cold plasma have been proposed as one of the most promising solutions for the food industry. This review will cover the current knowledge about the effects of cold plasma in polyphenols found in food products. The increasing number of studies in the last years supports the continuous search for specific treatment conditions for each type of food and the central role of plasma treatments as a food-processing technology.

## 1. Introduction

The increasing growth of the population pushes the food industry to increment its production to ensure quality and safety and, also, provide innovative foods [1]. Recently, the interest for foods rich in bioactive compounds has increased among consumers interested in enhancing their health (controlling blood cholesterol levels in hypercholesterolemic people by replacing conventional margarine with a cholesterol-lowering margarine, for instance) and well-being [2]. In order to attend this demand for products in this category, professionals in the food industry and researchers have been studying bioactive food components and developing technologies to preserve or improve their retention in food. Consequently, fruits, vegetables, and derived products were put in the spotlight as the main natural dietary sources of these bioactive compounds [3].

Polyphenols play a central role in this scenario, since these compounds are largely distributed in foods of vegetable origins and have been involved in the protection of human organs against inflammatory and neurodegenerative diseases and oxidative stress, the preservation of weight loss, and the positive influence in gut microbiota [4]. These compounds are considered as secondary metabolites of plant tissues and have a common structure composed of one aromatic ring with one or more hydroxyl groups [5]. The biosynthesis of polyphenols in plant tissues is affected by many factors, such as pathogens, herbivores, and ultraviolet (UV) radiation [6,7]. The polyketide pathway comprises the elongation of simple precursor molecules such as acetyl-coenzyme A and malonyl-coenzyme A (malonyl-CoA). The reaction occurs by successive condensations of malonyl-CoA-derived extender units into an activated acyl starter unit via the decarboxylative Claisen thioester mechanism. Due to the possibility of building the molecule backbone with malonyl-CoA-derived extender units of different degrees of oxidation, the pattern of elongation can be modified, and a vast range of molecules can be formed [8]. In the case of the shikimate acid pathway, the process starts with the condensation of phosphoenolpyruvic acid, which will produce crucial intermediates (such as shikimic and chorismic acid). Ultimately, this pathway generates polyphenols such as hydroxycinnamic acids [6].

It is important to mention that the current technologies used in the food industry are not entirely favorable to foods rich in polyphenols. The main strategy to improve the shelf life of food is centered in the thermal inactivation of microorganisms and enzymes. Due to the fact that polyphenols can be degraded by the high temperatures usually applied in food processing, the search for alternative approaches has increased in recent years [9]. In this sense, nonthermal technologies (particularly, plasma technology) are one of the best solutions to obtain both an increased shelf life [10,11] and maximize the retention of polyphenol in food products [12].

Due to the importance of plasma technology in vegetable food processing, this review aims to compile and discuss the recent findings and potential applications related to the use of plasma technology in uncut, fresh cut, and processed vegetables and fruits; beverages; and germinated seeds, with a special focus on the content and stability of polyphenols.

## 2. Fundaments, Main Applications, and Equipment Details of Plasma Technology

The plasma state is defined as the fourth state of matter. In other words, the matter displays a behavior different than that observed in the other three states. Plasma can be described as an electrically neutral gas with a meaningful portion of ionized particles. In this condition, the gas can be influenced by electric and magnetic fields and becomes an electrical conductor [13,14].

Plasma can be artificially generated by using a gas and adding some form of energy (electrical discharge, radiofrequency, and microwaves, for instance) [13]. Increasing the energy level of the system containing the gas causes the ionization of gas molecules by dissociation (diatomic gases such as N_2_ and O_2_). In this condition the gas begins to ionize and, also, remains electrically neutral (positive and negative charges from ions and electrons, respectively) [14]. Once the free electron reaches another molecule or atom, a chain reaction takes place. The chain reaction also disperses energy in the form of radiation (UV with wavelengths between 100 and 380 nm) [14]. The progression of gas ionization causes a significant reduction in the electrical resistance, and the gas becomes an electrical conductor, which causes an electrical discharge [13,14,15].

**Figure 1:**
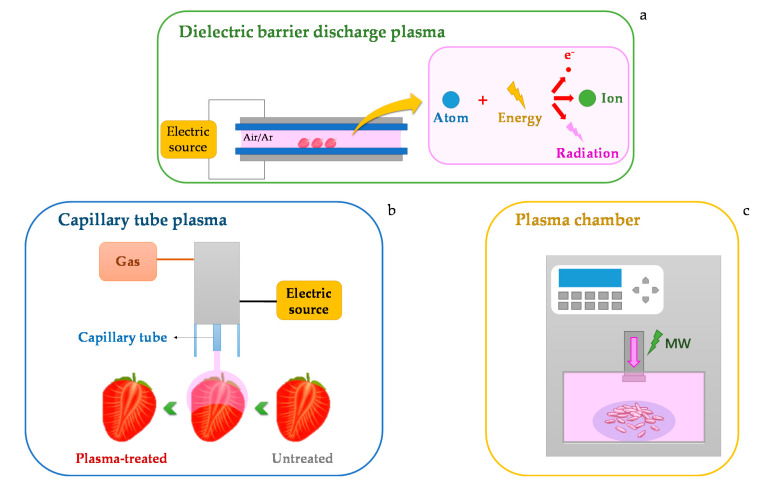
Schematic representation of the plasma equipment: (**a**) Dielectric barrier discharge; (**b**) Capillary tube plasma; (**c**) Plasma chamber. MW: microwave.

Some equipment used to apply plasma in foods of vegetable origins is presented in Figure 1. One of the equipments used to perform plasma treatments is the dielectric barrier discharge (DBD, Figure 1a). The equipment is comprised of two metal electrode plates (where at least one is covered by a dielectric layer) connected to an electric source [16,17]. In this equipment, the plasma formed between the plates due to the high potential difference between the plates is greater than the breakdown voltage. Another popular configuration to generate plasma is by a capillary tube (Figure 1b). The configuration of the equipment can be described as two concentric capillary electrodes. The external electrode is used to direct the plasma flow, and the inner electrode causes the ionization of the flowing gas due to the application of a high voltage from an electric source [18]. The plasma chamber is another relevant system (Figure 1c). In this case, the plasma is generated in a section prior to the chamber where ionization take place. Then, the plasma flows towards the chamber [19].

It is also relevant to mention that plasma treatments have been successfully used to reduce microbial contaminations in several types of products. The main mechanism of plasma technology to achieve reduced counts of pathogenic and spoilage microorganisms consist in the generation of reactive species—particularly, the reactive oxygen (ozone and O_2_^-^, for instance) and nitrogen (such as NO^•^) reactive species that cause pore formations and disruptions of the cellular membrane, irreversible DNA damage, the induction of apoptosis, and alterations of vital endogenous protein pathways [14,20]. Moreover, the decontamination effect of plasma technology has also been proven in food packaging materials. In studies with polyethylene, polyethylene terephthalate, polypropylene, and collagen casings, the plasma treatment reduced the counts of pathogenic bacteria and, also, did not influence the mechanical properties of the packaging materials [21,22,23].

Plasma technology has also been applied in the inactivation of enzymes. Taking into account that most food enzymes are proteins, the main mechanism to describe the inactivation of enzymes consists in the modification of the structural arrangement in the different levels (primary, secondary, and tertiary levels). The reactive species generated by plasma induced the loss of functional groups in side-chain amino acids such as _L_-valine into acetic and formic acids [24] and the loss of functional CNH_2_ and COOH groups from _L_-alanine [25] in the model system. In a similar way, the radical species generated by plasma treatments also induce the loss of secondary structures alfa-helix and beta-sheet, such as observed for polyphenol oxidase and peroxidase [26].

It is also important to mention that plasma technology has a great potential to be used as a surface treatment of food products, packages, and other materials used in food processing due to the low penetration of reactive species generated by plasma treatments into the foods or materials [27]. For instance, the penetration depths of hydroxyl radical, singlet oxygen, superoxide, and hydrogen peroxide are between 1 and 30 nm in liquid media. Moreover, the half-life of these species is limited to ns (hydroxyl radical) or ms (hydrogen peroxide) [27].

## 3. Effect of Plasma Treatment in Phenolic Compounds of Food

### 3.1. Uncut, Fresh Cut, and Processed Vegetables and Fruits

Recent studies point out that the phenolic compounds in raw (uncut food) and processed foods are less-influenced by plasma treatments (Table 1). For instance, the application of Ar plasma between 3 and 11 min on both fresh and dried (24 h at 36 °C) walnuts did not influence the phenolic contents (between 17.6 and 30.5 mg of gallic acid equivalent (GAE)/g dry weight (DW)) [28]. A similar outcome was obtained for strawberries treated with plasma generated by a dielectric barrier discharge [29]. In this study, the authors indicated that the anthocyanin content was not influenced by either the treatment time or the intensity of the voltage discharge. Likewise, the phenolic content of mandarin flesh treated with N_2_ plasma was not affected [30]. An interesting outcome was reported by the authors regarding the phenolic content on a mandarin peel. In this section of mandarin fruit, a slight increase was reported, indicating a physiological response to improve the protection against the radicals generated by the plasma treatment. However, this effect was noticed in the first day of storage and did not last until the end of storage (7 and 28 days at 25 and 4 °C, respectively).

In the study carried out by Lacombe et al. [31], the influence of the treatment time in the phenolic contents of uncut blueberries was evaluated. The authors observed a reduction in the phenolic contents in samples treated for longer periods, which was also associated with an increase in fruit temperatures (above 45 °C). This condition suggests that the longer exposure periods to radical species cause the degradation of phenolic compounds. The study carried out by Dong and Yang [32] indicated a nonsignificant effect on the anthocyanins of blueberries treated with DBD equipment. Moreover, the authors also reported an induction of polyphenol synthesis in the first days of storage by increasing the treatment time (up to 10 min). However, the additional phenolic content was gradually reduced during storage.

Matan et al. [33] indicated nonsignificant differences were obtained in the phenolic contents of slices of Dragon fruit treated by Ar plasma. However, an important outcome was reported by Ramazzina et al. [35], who studied the effects of plasma treatments on fresh-cut apples. In this experiment, the plasma treatment caused the reduction of some phenolic compounds: procyanidin dimer B2 and procyanidin B trimers. Another relevant outcome was the reduction on phenolic compounds from the hydrophobic fraction of the apple. Moreover, in both results, the decreasing effect was augmented by the treatment time (from 30 to 120 min).

In the case of the storage of fresh-cut samples, recent studies indicate high levels of polyphenols. The treatment of fresh-cut pitaya with plasma generated by dielectric barrier discharge equipment indicated an increase in the phenolic contents (particularly for gallic, protocatechuic, and *p*-coumaric acids) during storage (from 12 to 36 h at 15 °C) [36]. In a similar way, an experiment with fresh-cut strawberries displayed the same stimulatory effects during storage [17]. A significant increase in the contents of the total phenolic, anthocyanin, and flavonoid contents was observed during storage (4 °C), particularly on days 1, 3, and 5.

The use of a plasma treatment on onion powder did not influence the quercetin content during storage [19]. Interestingly, the study carried out by Thirumdas et al. [37] indicated that a plasma treatment increased the contents of phenolic compounds in basmati rice flour. In this experiment, the authors observed that the highest phenolic content was obtained by reducing the power from 40 to 30 W and treatment time from 10 to 5 min. Moreover, all plasma-treated showed higher phenolic contents than untreated basmati rice flour samples (0.48–0.53 vs. 0.44 mg GAE/100 g, respectively).

Collectively, the type of equipment and some processing variables deserve attention when carrying out plasma treatments in order to maximize the retention of polyphenols in uncut, fresh-cut, and processed vegetables and fruits. Regarding the equipment, more studies reported positive [17,32,36,37] and neutral [29] effects in polyphenols than negative [34,35] when the plasma treatment was carried out with DBD. In the case of capillary plasma equipment, neutral [28,33] and negative [31] effects of plasma treatments in these foods were reported. This outcome can be explained by the differences in the areas under the plasma, since DBD equipment can treat larger areas than capillary tube equipment [14,38]. This aspect was previously suggested to explain the differences in the inactivation of microorganisms between DBD and capillary plasma equipment on the surfaces of solid foods [14,38].

In the case of treatment time, the effect is particular for each sample, since both short (blueberries exposed for 120 s [31]) and longer (apple exposed for 120 min [34] and kiwi fruit for 40 min [35]) treatments can reduce the contents of polyphenols in uncut, fresh-cut, and processed vegetables and fruits. Differently, no clear effect was observed in the case of un-cut [28,29,30,31,32] vs. cut [17,33,34,35,36] samples, which suggests that this variable does not influence the use of plasma treatments. Therefore, scientific evidence indicates that further advances in uncut, fresh-cut, and processed vegetables and fruits can be made using DBD due to the larger treatment areas in comparison to capillary plasma, as indicated previously. At the same time, selecting the adequate treatment time for each matrix is also crucial to maximize the retention of the polyphenols.

### 3.2. Beverages Rich in Phenolic Compounds

The effects of plasma technology on the phenolic contents of juices, mixtures (with additional components than a conventional juice, such as functional beverages), and fermented beverages is shown in Table 2. Some studies indicate that the plasma treatment was associated with a reduction in the phenolic content. This outcome was reported for camu-camu (*Myrciaria dubia*) juice treated with a dielectric barrier discharge plasma [39]. In this experiment, the increasing of the excitation frequencies (from 200 to 960 Hz) induced a significant reduction in the anthocyanin content after the treatment. In a similar way, the increasing powers of plasma (from 30 to 50 W) on the processing of apple juice were associated with significant reductions in the total polyphenol content [40]. A similar reduction was reported for white grape juice exposed for up to 4 min to a plasma discharge [41]. The flavonoids and total phenolic content were reduced in comparison to fresh juice, regardless of the treatment time. Interestingly, the flavonol content increased as the treatment increased up to 4 min, which could indicate a degradation of the procyanidins (polymers of (epi)catechin).

Herceg et al. [42] compared the effects of plasma treatments with conventional pasteurization on the phenolic contents and predominant phenolic acids of pomegranate juice. The authors observed that both plasma-treated and pasteurized juices displayed higher polyphenol contents than fresh juice. Moreover, the highest increases were observed on ellagic acid (one of the main phenolic compounds in pomegranate juice) as the sample volume, gas flow, and treatment time were set to 4 cm^3^, 0.75 dm^3^/min, and 5 min, respectively. Additionally, the polyphenol profile of the pomegranate is composed of both simple and polymeric compounds (in addition to ellagic acid): gallic acid, protocatechuic acid, *p*-coumaric acid, caffeic acid, ferulic acid, chlorogenic acid, catechin, and punicalagin [42,52].

Likewise, the study carried out by Garofulić et al. [43] indicated that a plasma treatment improved the phenolic content of sour cherry in comparison to fresh and pasteurized juices. The authors concluded that increasing the volume sample to 3 mL and reducing the treatment time to 3 min was the most effective condition to improve the polyphenol content. In this scenario, the disruption of intact vacuoles (trapping the phenolic compounds) and the breakdown of chemical bounds between the polyphenols and structural molecules could explain the increase of the phenolic content.

The optimization of plasma treatments was studied by Paixão et al. [44] on siriguela (*Spondias purpurea* L.) juice. According to the authors, the highest phenolic content was obtained using a gas flow of 20 mL/min and treatment time of 15 min. Moreover, the phenolic content obtained from this optimal processing condition was superior to that obtained from fresh juice. The influence of the plasma treatment was also reported for cashew apple juice in a recent study [45]. Interestingly, the authors explored the influence of gas flow and treatment time and obtained different conditions to optimize the contents of anthocyanin (gas flow of 50 mL/min for 15 min) and the total polyphenols (gas flow of 30 mL/min for 10 min).

In the study performed by Mehta et al. [46], the contents of the selected phenolic compounds in a tomato-based beverage (tomato juice, coconut water, salt, sugar, beetroot juice, and sodium benzoate) was affected by the plasma treatment. While the contents of chlorogenic, sinapic, and gallic acids were improved by applying the plasma treatment for 10 min, no significant differences on the contents of these compounds was reported after 15 min of treatment. Similarly, application of the plasma treatment increased the contents of the phenolic compounds on a guava-flavored whey beverage (composed of guava pulp, milk, sugar, and gelatin powder) [47]. The authors obtained significantly higher phenolic contents with the plasma treatment than with pasteurization in this beverage (13.9–14.3 vs. 12.5 mg GAE/mL, respectively).

The use of plasma with increasing levels of oxygen (up to 1% in Ar) on blueberry juice was associated with high contents of phenolic compounds [48]. However, an inverse trend was observed for retention of the anthocyanin content and the content of oxygen in the gas composition. In another study with orange, tomato, apple, and sour cherry juices, the effects of the treatment time was evaluated [49]. In this case, the increase was mainly observed after 90 and 120-s treatments for all juices.

Although promising outcomes have been reported for juices, more complex beverages (particularly, functional and fermented beverages rich in polyphenols) show a more complex scenario for the utilization of plasma technology. The experiment carried out by Almeida et al. [50] explored the effects of direct (below the plasma discharge) and indirect (in the chamber but out of the plasma charge range) exposures of plasma discharges in a prebiotic orange beverage. The authors indicated that applying direct plasma did not influence the phenolic contents in functional beverages, whereas a reduction in the phenolic content was obtained by indirectly exposing the beverage to a plasma discharge. A recent study explored the effects of a plasma treatment on the phenolic contents of red and white wines from *Vitis vinifera* L. grapes [51]. The plasma treatment induced a reduction in the phenolic contents, particularly by depolymerizing procyanin B1 and B2 into epicatechin and catechin.

In the view of the studies about the influences of plasma treatments on polyphenols found in vegetable beverages, the selection of equipment, treatment time, and gas composition are of great importance to maximize the retention of polyphenols. The experiments carried out with capillary tube plasma displayed the most promising results [18,41,42,43,44,45,47,48,49]. Conversely, the same consideration cannot be done to DBD equipment [39,40,41,50]. In liquids, every volume element (including suspended particles bound to polyphenols) is exposed to the plasma discharge and the generated radicals, which makes the penetration depth of the radicals less relevant than indicated for solid samples [27]. Moreover, the contact between the plasma discharge and water in liquid foods causes the dissociation of water molecules and produces reactive oxygen species (ROS) that last for longer periods than the radicals generated from plasma itself [38]. The combination of these two factors with the small treating area of capillary plasma equipment [14,38] may explain, to some extent, the differences observed in polyphenols treated by DBD and capillary plasma equipment. In the case of treatment time and gas flow in capillary plasma equipment, the same trend was observed from several studies: a better preservation or enhanced content of polyphenols as these variables were increased [18,43,45].

The gas composition also plays an important role in the loss of polyphenols after a plasma treatment. It is worth remembering that the presence of O_2_ in the composition of the gas is of great importance to generate reactive species and active higher microbial inactivation levels [20]. In the same line of thought, the increase of the O_2_ proportion (intense generation of ROS) in the gas induced the consumption of polyphenols [48]. Interestingly, the same effect was not observed for beverages treated with N_2_ plasma [44,45,47]. This outcome could be explained by the reactive species generated (ROS vs. RNS), but further experiments are necessary to strengthen this hypothesis.

Therefore, it seems reasonable to indicate that further experiments in vegetable beverages rich in polyphenols should be carried out with capillary plasma equipment and considering the effects of gas flow and composition and treatment time to maximize the retention of polyphenols. It is also important to mention that the studies included in Table 2 display results after processing or within a short period of time and do not provide information about the effects of plasma treatments during storage. In this sense, major efforts are also necessary to clarify whether the higher contents of polyphenols are maintained. Additionally, the influence of temperature in the stability of polyphenols during storage should be evaluated.

### 3.3. Germinated Seeds

Germinated seeds and sprouts produced from broccoli, radishes, rice, and seeds are sources of macro- and micronutrients such as lipids; proteins; minerals; dietary fibers; and vitamins B1, B2, B3, B6, B9, C, and E that can be produced or catabolized during the germination stage of seeds [53,54]. Sprouts are also sources of bioactive compounds such as polyphenols, γ-aminobutyric acid, and isothiocyanates [53]. Moreover, the bioactive compounds found in broccoli sprouts have antihyperlipidemic and antihypertensive potentials [55]. Sprouts are usually consumed fresh or in minimally processed salads or side dishes, especially among health-conscious consumers [56]. However, the occurrence of foodborne outbreaks in recent years associated with the consumption of sprouts [57] generates a complex situation to ensure fresh-like sensory attributes, preserve bioactive compounds, and ensure safety [56]. The plasma treatment fits well in the processing of edible sprouts by fulfilling all these aspects [13,56]. Additionally, shortening the sprouting process and reducing the microbial load in seeds used in food crops is another potential application of plasma in food production [56].

Polyphenol biosynthesis takes place during the sprouting of seeds and can generate compounds from several classes, such as flavonoid, phenolic acids, proanthocyanidins, and ellagitannins [7]. Interestingly, the plasma treatment is a relevant technology to induce the germination in seeds that leads to an enhanced physiological response, reducing the surface contamination and increasing the water uptake towards sprouting [58]. Moreover, the biosynthesis of polyphenols is also stimulated and leads to an accumulation of these compounds due to the exposure of oxidant activity of radicals and seeds’ physiological responses [59].

Recent studies explored the effects of plasma treatments on the phenolic compounds prior to the sprouting of seeds (Table 3). In general, these studies indicate the absence of deleterious effects or an increase in the phenolic contents. For instance, the phenolic content of germinated broccoli seeds treated with plasma was not influenced, regardless of the treatment time (up to 3 min) [60]. In a similar way, Puligundla et al. [61] indicated similar phenolic contents between plasma-treated and control (not treated) germinated radish seeds. The effect of the treatment times (1, 2, and 3 min) was not significant in this study. Another recent study indicated the lack of a deleterious effect of a plasma treatment on germinated seeds (rapeseeds) [62].

Chen et al. [63] indicated that the polyphenol content of brown rice was influenced by the voltage of the plasma treatment. The authors observed that increasing the voltage applied on the plasma (range of 1–3 kV) improved the phenolic content of brown rice during germination, particularly after 18 h of imbibition. Similarly, Yodpitak et al. [64] observed that the content of the phenolic compounds of brown rice was improved by a plasma treatment during the germination period. According to the authors, the optimum values for power, gas flow, and treatment time were 135 W, 22 mL/min, and 75 s, respectively. However, a decay in the polyphenol contents over the germination period was observed for all treatments, regardless of the processing conditions.

Another relevant outcome about the use of plasma to improve the phenolic contents of germinated seeds was reported by Ji et al. [59] on *Coriandrum sativum* seeds. The authors studied the influence of time (1 and 3 min) and obtained a significant increase in the phenolic contents after applying the plasma on seeds for 1 min (germinated for two weeks), while no significant effect was observed after 3 min of the plasma treatments. It is important to mention that the plasma treatments were applied four times (once a day) prior to the germination period. In spinach seeds, the plasma treatment was also effective to induce the biosynthesis of the polyphenols [65]. In this food, the highest increase was obtained using N_2_ to produce the plasma and applying the treatment for 3 min. It is relevant to comment that the authors also studied the effects of the gas composition (N_2_ vs. air) on the phenolic contents and obtained opposing effects: while the samples treated with N_2_ displayed a general trend of increase in the polyphenol contents, a trend of reduction was reported for samples treated with air, regardless of the treatment times. This result highlights the importance of the gas composition on the polyphenols of seeds prior to germination.

In addition to the influences of the variables indicated previously, it is relevant to mention that the most successful outcomes on the polyphenols of germinated seeds were obtained after the treatment with DBD [63,64,65], whereas nonsignificant influences on the phenolic compounds were observed from germinated seeds treated with capillary tube equipment [60,61,62]. The main reason for this difference could be explained by differences in the treated areas between DBD and the capillary tube equipment (as indicated for the vegetable foods of Section 3.1) [14,38].

## 4. Influence of Plasma on Enzymes Related to the Biosynthesis and Degradation of Phenolic Compounds

Due to the nature of plasma, reactive species (ROS and/or RNS) can be generated and interact with the surfaces of foods. In this condition, a physiological response occurs. A complex antioxidant enzymatic system converts these reactive species into less harmful compounds for vegetable cells [66]. This response to a plasma treatment was reported by the significant increase in the activity and genes expression of ascorbate peroxidase, catalase, and superoxide dismutase during storage (seven days at 4 °C) in fresh-cut strawberries treated with plasma [17]. Dong and Yang [32] observed a similar effect on the superoxide dismutase SOD of blueberries treated with plasma and stored for 20 days at 25 °C.

In accordance with the activation of the physiological antioxidant system, the biosynthesis of phenolic compounds can be influenced by a plasma treatment. This scenario was reported in detail by Li et al. [17] in fresh-cut strawberries treated with plasma. The authors observed an intense generation of metabolites associated with the biosynthesis of phenylpropanoids; phenolic compounds (phenolic acids, flavones, and flavonols); and other metabolic processes. Moreover, the authors indicated the activity and gene expressions of enzymes involved in the biosynthesis of phenolic compounds from the phenylpropanoid pathway: cinnamate-4-hydroxylase, 4-coumarate coenzyme A ligase, and phenylalanine ammoniumlyase, which supports the influences of and provides an explanation, at least in part, for the stimulatory effects of plasma treatments in the biosynthesis of polyphenols in plant tissues. A similar outcome was reported in a recent study with fresh-cut pitaya (*Hylocereus undatus* Haw.) fruit [36].

Although the increased contents of phenolic compounds in edible plant tissues by plasma technology is a favorable aspect for the implementation of this technology in the food industry, the degradation of polyphenols must be considered. However, the knowledge about the mechanisms involved in the loss of anthocyanins are not fully understood [67]. In the enzymatic pathway, peroxidases, polyphenol oxidases, laccases, and lipoxygenases (naturally found in vegetable foods) have been suggested to cause the oxidative degradation of phenolic compounds in vegetable foods [68].

Plasma treatments can inactivate food enzymes by causing changes in the secondary structures (increasing the β-sheet and reducing the α-helix structures) of polyphenol oxidases and peroxidases [69]. Some recent studies that explored the uses of plasma treatments in phenolic compounds indicate contrasting results about the association of these enzymes with the degradation of phenolic compounds in food products. For instance, de Castro et al. [39] indicated that the anthocyanin content, polyphenol oxidase, and peroxidase activities displayed similar reductions as the power of the plasma treatment was increased in camu-camu juice. In the case of siriguela juice, the plasma treatment caused different effects on polyphenol oxidase and peroxidase [44]. While the polyphenol oxidase activity was reduced, the peroxidase activity was slightly improved. Additionally, the phenolic content of the plasma-treated juice was improved in comparison to the untreated samples. In the context of polyphenol stability, the inactivation of polyphenol oxidase indicated by the plasma treatments in these studies is an important outcome to prevent enzymatic oxidation and the loss of polyphenols.

## 5. Conclusions

The influence of plasma technology in the phenolic content of vegetable foods is dependent on the equipment configurations and treatment conditions. In solid foods (such as fresh vegetables and fruits and germinated seeds), DBD is the most suitable choice to maximize the retention of polyphenols, while capillary tube equipment is suitable to reduce the loss of polyphenols in liquid foods. The appropriate application of plasma technology to achieve suitable preservations and conservations of biological potential can be seen as a delicate balance by an abiotic stress (reactive species) in foods of vegetable origins. Plasma treatments can rupture particles, entrapping the phenolic compounds in both solid and liquid vegetable foods, as well as stimulating the antioxidant defenses in germinated seeds. It is also relevant to mention that exposing foods to plasma can be deleterious to phenolic compounds, once the intensity of the treatment overcomes the plant or food’s capacity to prevent oxidative reactions or causes the degradation of polyphenol molecules (oxidation response to reactive species or depolymerization, for instance).

Finally, it seems reasonable to indicate that the processing conditions must be optimized, considering the composition of the gas, the mechanism to generate plasma (DBD and capillary equipment), the preparation of the plant-based food (solid or liquid), the main phenolic compounds (simple or polymeric), and the inactivation of the polyphenolic degradative enzymes, as well as standardizing the variables selected for the plasma treatments of food to facilitate comparisons among studies. Further experiments should also explore the progression towards industrial applications by optimizing the plasma treatment from batch to continuous systems, which can strengthen the role of plasma technology among nonthermal and thermal technologies.

## Figures and Tables

**Table 1:** Influence of plasma treatments on phenolic compounds of uncut, fresh cut, and processed foods.

Food	Gas (Applier and Energy Source)	Treatment Conditions	Point(s) of Assay	Effect of Plasma Treatment on Phenolic Compounds	Reference
Fresh and dried walnuts (uncut)	Ar (capillary tube, electric source)	Voltage (15 kV); frequency (12 kHz); gas flow (1 L/min); and time (3, 5, 7, 9, and 11 min)	After treatment	No effect of treatment time or storage time on TPC	[28]
Strawberries (uncut)	Air (dielectric barrier discharge, electric source)	Voltage discharge (60 and 80 kV) and time (1 and 5 min)	After treatment	No effect on anthocyanin content	[29]
Strawberries (fresh cut)	Air (dielectric barrier discharge, electric source)	Voltage (45 kV) and time (1 min)	7 days at 4 °C	Increase TPC, flavonoid, and anthocyanin contents up to day 5	[17]
Mandarin (uncut)	N_2_, (chamber, MW source)	MW power (900 W), frequency (2.45 GHz), vacuum, and time (10 min)	7 at 25 °C and 28 days at 4 °C	No effect on TPC of flesh; slight increase on peel	[30]
Blueberries (uncut)	Air (capillary tube, electric source)	Power (549 W), frequency (47 kHz), gas flow (4 ft^3^/m), and time (15–120 s)	After treatment	Reduction of anthocyanin content as treatment time increased	[31]
Blueberry (uncut)	Air (dielectric barrier discharge, electric source)	Voltage (36 V), current (1.8 A), and time (up to 10 min)	20 days at 25 °C	Highest anthocyanin levels were obtained using 6 and 8 min; effect lasted for 20 days	[32]
Dragon fruit (fresh cut)	Ar (capillary tube, RF source)	RF power (40 W) and time (60 s)	After treatment	No effect on TPC	[33]
Apples (fresh cut)	Air (dielectric barrier discharge, electric source)	Power (150 W), frequency (12.7 kHz), gas flow (1.5 L/min), and time (30 and 120 min)	After treatment	Reduced TPC; reduction of some procyanidin dimers and trimers (120 min)	[34]
Kiwi (fresh cut)	Air (dielectric barrier discharge, electric source)	Voltage (15 kV) and time (20 and 40 min)	4 days at 10 °C	No effect on hydrophilic fraction of phenolics; slight reduction on hydrophobic fraction of phenolics after 4 days	[35]
Pitaya (fresh cut)	Air (dielectric barrier discharge, electric source)	Voltage (60 kV) and time (5 min)	48 h at 15 °C	Slight increase on selected phenolic and expression of genes related to polyphenol synthesis during storage	[36]
Onion powder	He (chamber, MW source)	MW intensity (400 W), frequency (2.45 GHz), gas flow (1 L/min), pressure (0.7 kPa), and time (40 min)	28 days at 4 and 25 °C	No effect on quercetin content	[19]
Basmati rice flour	Air (dielectric barrier discharge, RF source)	RF power (30 and 40 W), frequency (13.56 MHz), and time (5 and 10 min)	After treatment	Increase TPC content by reducing time and power	[37]

Ar: argon, He: helium, N_2_: nitrogen, TPC: total phenolic content, MW: microwave, and RF: radio frequency.

**Table 2:** Influences of plasma treatments on the polyphenols of beverages rich in phenolic compounds.

Food	Gas (Applier and Energy Source)	Treatment Conditions	Effect	Reference
Camu-camu juice	Air (dielectric barrier discharge, electric source)	Frequency (200–960 Hz) and time (15 min)	Reduced phenolic and monomeric anthocyanin contents as frequency was improved	[39]
Apple juice	Air (dielectric barrier discharge, electric source)	Power (30, 40, and 50 W) and time (40 s)	Reduction on TPC as the power increased	[40]
White grape juice	Air (dielectric barrier discharge, electric source)	Voltage (80 kV) and time (1–4 min)	Reduced TPC and flavonoid contents; increased flavonol	[41]
Pomegranate juice	Ar (capillary tube, electric source)	Power (4 W); sample (3, 4, and 5 cm^3^); gas flow (0.75, 1, and 1.25 dm^3^/min); and time (3, 5, and 7 min)	Increased TPC similarly to pasteurization	[42]
Sour cherry Marasca juice	Ar (capillary tube, electric source)	Power (4 W); sample (2, 3, and 4 mL); gas flow (0.75, 1, and 1.25 L/min); and time (3, 4, and 5 min)	Increased anthocyanin and TPC	[43]
Siriguela juice	N_2_ (capillary tube, radiofrequency source)	Gas flow (10, 20, and 30 mL/min) and time (5, 10, and 15 min)	Increased TPC	[44]
Cashew apple juice	N_2_ (capillary tube, radiofrequency source)	Gas flow (10, 30, and 50 mL/min) and time (5, 10, and 15 min)	Increasing effect was dependent of compound class	[45]
Tomato-based beverage	Air (dielectric barrier discharge, electric source)	Voltage (60 kV), frequency (50 Hz), and time (10 and 15 min)	Increased TPC and individual polyphenols using treatment for 10 min	[46]
Guava-flavored whey beverage	N_2_ (capillary tube, radiofrequency source)	Power (400 W); frequency (50 kHz); gas flow (10, 20, and 30 mL/min); and time (5, 10, and 15 min)	Higher phenolic content than pasteurized sample	[47]
Blueberry juice	Ar and O_2_ (capillary tube, electric source)	Voltage (11 kV); frequency (1000 Hz); O_2_ content (0%, 0.5%, and 1%); and time (2, 4, and 6 min)	Increased TPC as treatment time was increased and O_2_ content in gas was reduced	[48]
Orange, tomato, apple, and sour cherry juices	Dry air (capillary tube, electric source)	Frequency (25 kHz), power (650 W), and time (30–120 s)	The highest increase was obtained with 90 and 120 s	[49]
Prebiotic orange juice	Air (dielectric barrier discharge, electric source)	Voltage (70 kV), frequency (50 Hz), exposure (direct or indirect), and time (15–60 s)	No effect after direct exposure treatment; indirect exposure induced the loss of phenolic compounds as treatment time increased	[50]
Red and white wines	Ar (capillary tube inserted in liquid, electric source)	Gas flow (4 L/min); frequency (60, 90, and 120 Hz); and time (3, 5, and 10 min)	Reduced TPC, anthocyanin, and tannin contents as frequency and time were increased	[51]

Ar: argon, N_2_: nitrogen, O_2_: oxygen, and TPC: total phenolic content.

**Table 3:** Influence of the plasma treatments on phenolic compounds of sprouting and sprouted foods.

Food	Gas (Applier and Energy Source)	Treatment Conditions	Point(s) of Assay	Effect of Plasma Treatment on Phenolic Compounds	Reference
Broccoli seeds (*Brassica oleracea* var. kialica plen. Mill.)	Air (capillary tube, electric source)	Voltage (20 kV); frequency (58 kHz); and time (1, 2, and 3 min)	24 h at 25 °C	No significant effect	[60]
Radish seeds (*Raphanus sativus* L.)	Air (capillary tube, electric source)	Voltage (20 kV); current (1.5 A); frequency (58 kHz); and time (1, 2, and 3 min)	4 days at 25 °C	No significant effect	[61]
Rapeseed seeds (*Brassica napus* L.)	Air (capillary tube, electric source)	Voltage (20 kV); frequency (58 kHz); and time (1, 2, and 3 min)	4 days at 25 °C	No significant effect	[62]
Brown rice (*Oryza sativa* L. var. Koshihikari)	Air (dielectric barrier discharge-like apparatus, electric source)	Voltage (1, 2, and 3 kV); current (1.2 mA); and plasma time (10 min)	12, 18, and 24 h at 25 °C	Increased TPC using 2 and 3 kV after 18 h of imbibition	[63]
Brown rice (*Oryza sativa* L.)	Ar (dielectric barrier discharge, RF source)	Power (100–200 W), gas flow (18–24 mL/min), and time (25–300 s)	4 days at 25–28 °C	Anticipated and increased the rise of TPC during germination	[64]
*Coriandrum sativum* L. seeds	N_2_ (capillary tube, MW source)	Power (400 W), frequency (2.45 GHz), gas flow (10 L/min), and time (1 and 3 min)	2 and 4 weeks	Highest increased was obtained after 1 min	[59]
Spinach seeds (*Spinacia oleracea* L.)	N_2_ and air (dielectric barrier discharge, electric source)	Voltage (6 kV), current (14 mA), gas flow (1.5 L/min), and time (up to 5 min)	5 weeks	Highest increase was obtained using N_2_ for 3 min	[65]

Ar: argon, N_2_: nitrogen; TPC: total phenolic content; MW: microwave, and RF: radio frequency.
